# Diversity and Stability of Lactic Acid Bacteria in Rye Sourdoughs of Four Bakeries with Different Propagation Parameters

**DOI:** 10.1371/journal.pone.0148325

**Published:** 2016-02-05

**Authors:** Ene Viiard, Marianna Bessmeltseva, Jaak Simm, Tiina Talve, Anu Aaspõllu, Toomas Paalme, Inga Sarand

**Affiliations:** 1 Competence Center of Food and Fermentation Technologies, Tallinn, Estonia; 2 Department of Food Processing, Tallinn University of Technology, Tallinn, Estonia; 3 Centre for Biology of Integrated Systems, Tallinn University of Technology, Tallinn, Estonia; 4 Department of Gene Technology, Tallinn University of Technology, Tallinn, Estonia; University of Naples Federico II, ITALY

## Abstract

We identified the lactic acid bacteria within rye sourdoughs and starters from four bakeries with different propagation parameters and tracked their dynamics for between 5–28 months after renewal. Evaluation of bacterial communities was performed using plating, denaturing gradient gel electrophoresis, and pyrosequencing of 16S rRNA gene amplicons. *Lactobacillus amylovorus* and *Lactobacillus frumenti* or *Lactobacillus helveticus*, *Lactobacillus pontis* and *Lactobacillus panis* prevailed in sourdoughs propagated at higher temperature, while ambient temperature combined with a short fermentation cycle selected for *Lactobacillus sanfranciscensis*, *Lactobacillus pontis*, and *Lactobacillus zymae* or *Lactobacillus helveticus*, *Lactobacillus pontis* and *Lactobacillus zymae*. The ratio of species in bakeries employing room-temperature propagation displayed a seasonal dependence. Introduction of different and controlled propagation parameters at one bakery (higher fermentation temperature, reduced inoculum size, and extended fermentation time) resulted in stabilization of the microbial community with an increased proportion of *L*. *helveticus* and *L*. *pontis*. Despite these new propagation parameters no new species were detected.

## Introduction

Sourdough is a mixture of flour and water that is fermented with lactic acid bacteria (LAB) and yeasts. Traditional sourdoughs are propagated by backslopping over many decades by mixing a portion of mature sourdough with fresh flour and water and fermenting this into a new batch of sourdough [1, 2]. In mature sourdough, both homo- and hetero-fermentative species of LAB are prevalent and the community is typically dominated by members from the genus *Lactobacillus* [3].

Over 50 different species of LAB have been isolated from sourdoughs of different origin [4]. Despite this large number of identified species, mature sourdoughs typically contain only two or three dominant species. *Lactobacillus brevis*, *Lactobacillus fermentum*, *Lactobacillus plantarum*, *Lactobacillus sanfranciscensis*, and *Lactobacillus acidophilus* [1, 5–6] species are most often encountered in rye sourdoughs, which are used to make rye bread, a staple of the Nordic diet.

Sourdoughs can be classified into three types according to the technology used for their production [1, 7–8]. Type I sourdoughs are produced using a traditional method based on daily renewal. Type II sourdoughs used in large-scale production are semi-fluid and have good handling properties. Long term continuous propagations are common in type II sourdough processes. Type III sourdoughs are generally initiated by starter cultures and are dried before use. These sourdoughs are commonly used as taste and aroma enhancers.

A sourdough cycle can be started by either the spontaneous fermentation of flour, inoculation with mature sourdough, or with a starter culture [2]. The baking industry currently tends to begin sourdough fermentation with defined commercial starter cultures with specific properties [1]. Unfortunately, those strains may not adapt to the sourdough propagation conditions in the bakery and are often not competitive enough in the long term to fight off LAB that enter the process from either raw materials or the bakery environment. Therefore, to maintain a desirable microbial community, the sourdough cycle is frequently restarted [9–10]. The microbial composition of sourdoughs is affected by the process technology and applied conditions: fermentation temperature and time, inoculum size, water content (characterized by dough yield), production environment, and type of flour [1, 11–13].

Information regarding the composition and stability of sourdoughs used in industrial-scale production is limited. The traditions of sourdough preparation and bread making are region-dependent, which influences the sensory characteristics chosen for industrially prepared breads [14]. The aims of this work are i) to compare LAB communities in sourdoughs that originate from bakeries that apply different sourdough propagation parameters and ii) to evaluate the stability of these microbial communities and their influence on the chemical characteristics of the sourdough over many months of daily renewal. Both culture dependent and culture independent methods were used to characterize the microbial communities within the sourdough samples.

## Materials and Methods

### Sourdough samples from bakeries

The sourdoughs studied originate from four bakeries that use flour of the same origin (rye flour type 1370, extraction rate 85%, Tartu Mill AS, Estonia) and are referred to as A_bakery_, B_bakery_, C_bakery_, and D_bakery_. The bakeries use different sourdough propagation parameters (presented in Table 1); two sourdoughs are type II (A_bakery_ and B_bakery_) and two are type I (C_bakery_ and D_bakery_). In A_bakery_ and B_bakery_ the sourdough fermentation temperature was precisely controlled while C_bakery_ and D_bakery_ propagated sourdoughs at room temperature which fluctuated seasonally. The temperature of each D_bakery_ sourdough sample is provided in Table 1.

**Table 1:** Sourdough propagation parameters (fermentation temperature and time, inoculum size, dough yield), starter used to initiate fermentation, and sampling schedule in four Estonian bakeries (A_bakery_, B_bakery_, C_bakery_ and D_bakery_). In C_bakery_ and D_bakery_ the fermentation was carried out at room temperature (RT). ‘Months’ indicate time passed from the beginning of a new sourdough cycle (A_bakery_, B_bakery_, and C_bakery_) or from the collection of the first sourdough sample (D_bakery_).

Bakery	A_bakery_	B_bakery_	C_bakery_	D_bakery_
Fermentation temperature	32°C	42–44°C	RT (up to 28°C)	RT (19–30°C)
Inoculum size	10%	6%	~ 33%	~ 33%
Fermentation time	10 h	16 h	~ 4 h	~ 4 h
Dough yield	250	400	~ 200	~ 200
Starter	Freeze-dried sourdough culture	Active sourdough starter	Commercial freeze-dried starter	Commercial freeze-dried starter
Analyzed samples	A0 –freeze-dried sourdough	Bs– 3 years propagated sourdough	C0 –freeze-dried commercial starter	D1 –approx. 30 years propagated sourdough (ambient temperature 19°C)
	A1–1.2 months	B0 –fresh sourdough starter	C1–12 months	D2–3 months (30°C)
	A2–3.5 months	B1–0.25 months	C2–21 months	D3–5 months (28°C)
	A3–4.5 months	B2–1 month	C3–28 months	D4–19 months (23°C, before transfer to 30°C)
	A4–8.5 months	B3–2.5 months		D5–24 months (5 months after transfer to 30°C)
		B4–6 months		

The sourdough process in A_bakery_ was initiated with a freeze-dried sourdough made from a mature sourdough produced six years previously at the same bakery (sample A0). B_bakery_ sourdough had been successfully propagated for three years (sample Bs) and was then renewed from fresh cooled sourdough sourced from another bakery belonging to the same corporation (sample B0). C_bakery_ sourdough fermentation was initiated with a freeze-dried commercial starter (C0) one year before the first sourdough sample was collected. D_bakery_ sourdough was initiated in the 1980’s from a commercial starter. During the course of this study D_bakery_ adopted new propagation parameters with a well-controlled fermentation temperature in an attempt to improve both the stability of the sourdough and optimize the sourdough production cycle (Table 1). The cycle was extended from 4 h to 12 h and the fermentation temperature was increased to 30°C. In addition, the inoculum size was lowered from approximately 33% to 10%. Sourdough sample D4 was taken before adopting the new sourdough propagation cycle. Sample D5 was collected from sourdough that had been propagated for five months after the upgrade. In addition, 18 sourdough samples were collected between these two time points, however, only pH and DGGE analysis was performed.

### Chemical analysis of sourdoughs

The pH and total titratable acidity (TTA) values of each sourdough sample were measured in triplicate. For each analysis 5 g of sourdough was homogenized with 45 ml of distilled water. The pH and TTA were measured with Food and Beverage Analyzer D22 (Mettler-Toledo International Inc., USA). TTA is given as ml of 0.1 N NaOH used to titrate 10 g of sourdough sample to pH 8.5.

### Enumeration of lactic acid bacteria

The cell density of culturable LAB in each sourdough sample was determined by plate counting. 5 g of sourdough was mixed with 45 ml of sterile 0.85% NaCl solution. A series of decimal dilutions were plated on MRS agar (LabM, UK) in duplicate. In addition, sample C3 was plated on an mMRS agar (MRS with added 2% maltose; pH 5.6) and SDB agar (2% maltose; 0.03% Tween80; 0.6% trypticase; 1% yeast extract; pH 5.6) [15]. Plates from A_bakery_, C_bakery_ and D_bakery_ were incubated at 30°C, while those from B_bakery_ were incubated at 42°C; all under anaerobic conditions. A BD GasPak EZ System (Becton Dickinson Microbiology Systems, USA) was used to maintain an anaerobic environment.

### DNA extraction from lactic acid bacteria isolates and sourdough samples

Selected colonies were checked for purity by streaking. The cultures were suspended in dH_2_O with a loop and subjected to DNA extraction using FTA membrane cards (Whatman Inc., USA) following the method provided by the manufacturer. Total DNA extraction from the sourdough samples was performed using 5 g of sourdough, which had been homogenized by vortexing with 45 ml of sterile physiological solution. This suspension was then centrifuged at 4°C for 5 minutes at 1000 × g. The supernatant was collected and centrifuged at 4°C for 15 minutes at 5000 × g. A GenElute Bacterial Genomic DNA Kit (Sigma-Aldrich, Inc., USA) was used for DNA extraction from the obtained pellet in the case of B_bakery_, C_bakery_, and D_bakery_ according to the manufacturer’s instructions. A modified phenol-chloroform extraction was used for samples taken from A_bakery_ [16].

### Fingerprint typing of isolates with Rep-PCR

For each sourdough sample, 20 colonies were randomly picked from plates with suitable dilution (usually 20 to 200 colonies per plate) and analyzed by Rep-PCR. Rep-PCR with primer (GTG)_5_ (all primers in this work were obtained from Microsynth, Switzerland) was performed as described by Viiard [16] with slight modification: all PCR components were sourced from Solis BioDyne (Estonia). Share of each LAB fingerprint type within selected isolates was calculated as a percentage of the colonies analyzed.

### Identification of lactic acid bacteria by 16 rRNA gene sequence analysis

One to two representatives of each fingerprint type group detected using Rep-PCR were selected for 16S rRNA gene analysis. 16S rRNA gene fragments were amplified using the universal primers 27f-YM [17] and 16R1522 [18] followed by column purification of the amplified fragment with a GeneJET PCR Purification Kit (Thermo Scientific Inc., USA) and sequenced in a commercial facility. The partial 16S rRNA gene sequences obtained (approximately 700 bp) were searched against GenBank database using the BLAST algorithm (Basic Local Alignment Search Tool, National Center for Biotechnology Information, USA) and the results were confirmed with the Greengenes 16S rRNA gene database (Lawrence Berkeley National Laboratory, USA).

### Denaturing gradient gel electrophoresis analysis of microbial communities

Denaturing gradient gel electrophoresis (DGGE) analysis was performed to monitor the dynamics of microbial communities within sourdoughs. V3 region of the bacterial 16S rRNA genes was amplified using primers F357-GC and 518R as described by Gafan and Spratt [19] to obtain approx. 160 bp fragments. Polyacrylamide gel (8% acrylamide-N,N’-methylenebisacrylamide; 37.5:1) with a gradient from 35 to 70% urea and formamide (100% corresponding to 40% formamide and 7 M urea) was used. Electrophoresis was performed with an INGENY phorU (Ingeny BV International, The Netherlands) at a constant voltage of 70 V at 60°C for 17 h. For yeasts, the primers U1GC and U2 were used to amplify the 28S rRNA genes, as described by Meroth [20] to obtain approx. 300 bp amplicons. A gradient of 30–50% was used and electrophoresis was performed at a constant voltage of 130 V at 60°C for 4.5 h.

The gels were stained with ethidium bromide and digitized using an ImageQuant 400 system (GE Healthcare, USA). Bands of interest were excised and DNA within these bands was eluted by incubation in a TE buffer overnight at 4°C. The eluted fragments were reamplified using F357/518R or U1/U2 primer pairs for bacterial and yeast DNA, respectively, and sequenced in a commercial facility.

### Pyrosequencing of bar-coded 16S rRNA gene amplicons

Universal primers 8F and 357R were used for PCR amplification of the V2–V3 hypervariable regions of 16S rRNA genes [21–22]. The amplicon mixtures were pyrosequenced using a 454 GS FLX+ System (Roche 454 Life Sciences, USA). The 454 pyrosequencing data was processed using MOTHUR v.1.32.1 [23] according to standard operating procedures [24]. Reads shorter than 150 bp or containing more than eight homopolymers were removed from the dataset. Sequences were aligned to the SILVA reference 16S rRNA database [25]. Operational Taxonomic Units (OTUs) were defined using an average neighbor clustering algorithm based on 97% sequence identity. Normalized OTU counts at 500 reads were calculated with the R (version 3.0.3) software package “vegan” version 2.0–10. In addition we calculated the rate of forming new OTUs when one sequence is added to the set of 500 sequences. The closest match to each OTU within the Greengenes 16S rRNA gene database (Lawrence Berkeley National Laboratory, USA) was identified using BLAST with a minimum of 97% similarity. The relative abundance of OTUs was calculated as the number of sequences for each OTU divided by the total number of bacterial sequences obtained for each sourdough sample. To estimate the beta-diversity, non-metric multidimensional scaling (NMDS) was conducted using Yue and Clayton distances [26] within MOTHUR and results were visualized using R software (version 3.0.3).

### Nucleotide sequence accession numbers

Pyrosequencing data is available in the GenBank database under accession numbers KM972414—KM972548.

## Results

### Chemical analysis and LAB enumeration of rye sourdoughs

The chemical properties of A_bakery_, B_bakery_ and C_bakery_ sourdoughs were rather stable throughout the study (Table 2). The average pH value of the sourdough throughout the study was 3.64 ± 0.08 in A_bakery_, 3.56 ± 0.08 in B_bakery_ and 4.10 ± 0.09 in C_bakery_. B_bakery_ sourdough was characterized by a high TTA in all samples except the initiating starter dough B0. Significant seasonal fluctuations in pH occurred in the sourdough from D_bakery_, where the average pH value of the sourdough was 4.06 ± 0.18. Sample D1 with a pH value of 4.28 was taken in February, when temperature of the sourdough was 19°C. During warmer months June (D2) and August (D3) the pH values decreased to 3.96 and 3.86, respectively.

**Table 2:** Mean values ± standard deviation of pH, total titratable acidity (TTA) and cell density of presumptive lactic acid bacteria (LAB) of rye sourdoughs from four Estonian bakeries (A_bakery_, B_bakery_, C_bakery_ and D_bakery_). Samples are coded according to the description reported in Table 1.

Sample	pH	TTA	LAB
		(ml 0.1 N NaOH / 10 g)	(log CFU g^-1^)
A0	NA*	NA	7.08 ± 0.11
A1	3.67 ± 0.06	22.30 ± 0.56	8.82 ± 0.07
A2	3.71 ± 0.01	18.89 ± 0.05	9.04 ± 0.06
A3	3.63 ± 0.04	21.30 ± 0.41	8.84 ± 0.07
A4	3.53 ± 0.02	21.84 ± 0.30	9.08 ± 0.03
Bs	3.60 ± 0.05	31.33 ± 0.13	8.63 ± 0.16
B0	3.40 ± 0.12	21.60 ± 0.58	8.56 ± 0.14
B1	3.63 ± 0.08	30.23 ± 0.02	8.11 ± 0.06
B2	3.57 ± 0.06	31.38 ± 1.27	8.94 ± 0.08
B3	3.57 ± 0.05	34.51 ± 1.28	8.93 ± 0.24
B4	3.58 ± 0.06	33.18 ± 1.09	8.85 ± 0.05
C0	NA	NA	6.95 ± 0.04
C1	4.11 ± 0.09	16.50 ± 1.11	6.56 ± 0.07
C2	4.00 ± 0.07	18.20 ± 0.03	6.64 ± 0.01
C3	4.18 ± 0.11	17.10 ± 0.58	8.28 ± 0.03
D1	4.28 ± 0.06	16.94 ± 1.08	8.00 ± 0.03
D2	3.96 ± 0.16	18.30 ± 1.06	8.80 ± 0.08
D3	3.86 ± 0.06	23.19 ± 1.10	9.05 ± 0.12
D4	4.12 ± 0.11	17.85 ± 0.03	8.01 ± 0.11
D5	3.78 ± 0.07	18.98 ± 0.03	8.31 ± 0.08

* NA–not acquired

The cell density of LAB was high and stable in A_bakery_ (on average 8.95 ± 0.13 log CFU g^-1^) and B_bakery_ (on average 8.67 ± 0.32 log CFU g^-1^), with the exception of sample B1, which exhibited a lower cell density (Table 2). In case of C_bakery_ unusually low values of LAB cell density (order of magnitude: 6 log CFU g^-1^) were obtained for samples C1 and C2 (Table 2). Two fold higher cell density was found for sample C3, but the related plates were incubated for additional 24 h compared to the samples C1 and C2. In D_bakery_ the LAB cell density (on average 8.47 ± 0.54 CFU g^-1^) depended on the ambient temperature in the bakery and was higher during summer (samples D2 and D3).

### Analysis of the LAB community in rye sourdoughs

**Fig 1:**
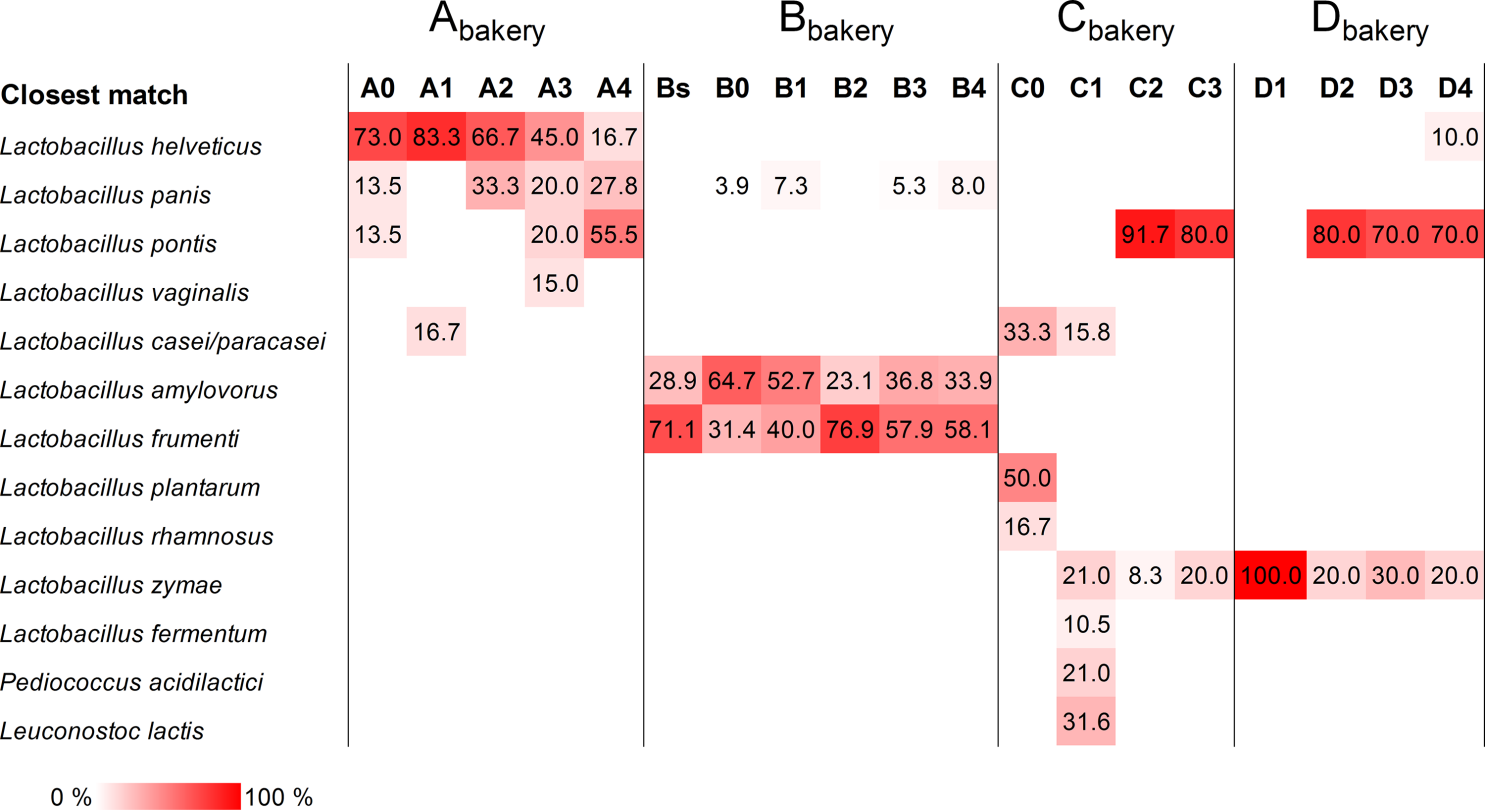
Composition of lactic acid bacterium species, expressed in percentage of the total number of isolates, in rye sourdoughs from four Estonian bakeries (A_bakery_, B_bakery_, C_bakery_ and D_bakery_). Samples are coded according to the description reported in Table 1.

**Fig 2:**
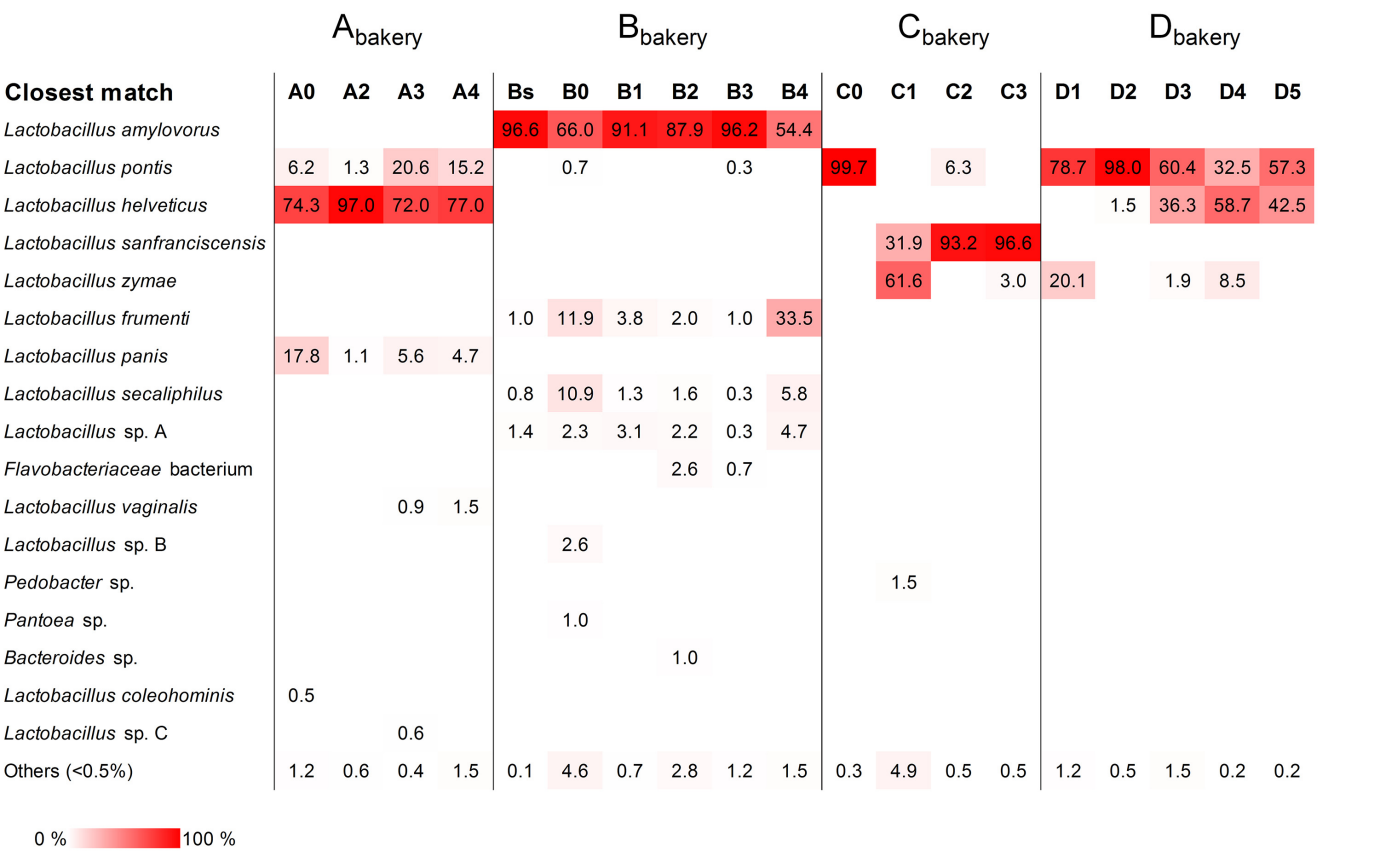
Relative abundance of bacterial species/genera detected in rye sourdoughs from four Estonian bakeries (A_bakery_, B_bakery_, C_bakery_ and D_bakery_) using pyrosequencing of 16S rRNA gene amplicons. Samples are coded according to the description reported in Table 1.

Sourdough samples from A_bakery_ were monitored for over eight months after renewal from a freeze-dried starter (previously published by Viiard [16]). Based on the results from culture dependent analysis, the dominating LAB in the freeze-dried starter dough belonged to species *Lactobacillus helveticus*, *Lactobacillus panis* and *Lactobacillus pontis* (Table 3 and Fig 1). It was shown that during continuous propagation of sourdough in A_bakery_ the proportion of *L*. *helveticus* colonies decreased, and that of *L*. *panis* and *L*. *pontis* increased. DGGE analysis, however, revealed *L*. *helveticus* as a prevalent species during over eight months of propagation (Table 3 and S1 Fig). The pyrosequencing analysis confirmed that the microbial community within the A_bakery_ sourdough was remarkably stable (Table 3 and Fig 2). Data of sequences and OTUs from 16S rRNA pyrosequencing performed using DNA extracted from sourdoughs sampled at A_bakery_, B_bakery_, C_bakery_ and D_bakery_ are shown in Table 4. The trimmed amplicon length of all sourdough samples was in the range 228–262 bp. The total number of sequences before processing (raw reads) was 42,388; on average 2231 sequences per sample were obtained. After data processing (reads) in total 34,906 sequences remained.

**Table 3:** Bacterial species/genera found in the rye sourdough samples from four Estonian bakeries (A_bakery_, B_bakery_, C_bakery_ and D_bakery_) through culture dependent analysis, DGGE or 16S pyrosequencing. Presence (+) or absence (‒) of species is indicated for each sample in the following order: culture dependent analysis / DGGE / 16S pyrosequencing. N–not analyzed. Samples are coded according to the description reported in Table 1.

		*Bacteroides* sp.	*Flavobacteriaceae bacterium*	*Lactobacillus amylovorus*	*Lactobacillus casei/paracasei*	*Lactobacillus coleohominis*	*Lactobacillus fermentum*	*Lactobacillus frumenti*	*Lactobacillus helveticus*
A_bakery_	A0	‒/‒/‒	‒/‒/‒	‒/‒/‒	‒/‒/‒	‒/‒/+	‒/‒/‒	‒/‒/‒	+/+/+
	A1	‒/‒/‒	‒/‒/‒	‒/‒/‒	+/‒/‒	‒/‒/‒	‒/‒/‒	‒/‒/‒	+/+/+
	A2	‒/‒/‒	‒/‒/‒	‒/‒/‒	‒/‒/‒	‒/‒/‒	‒/‒/‒	‒/‒/‒	+/+/+
	A3	‒/‒/‒	‒/‒/‒	‒/‒/‒	‒/‒/‒	‒/‒/‒	‒/‒/‒	‒/‒/‒	+/+/+
	A4	‒/‒/‒	‒/‒/‒	‒/‒/‒	‒/‒/‒	‒/‒/‒	‒/‒/‒	‒/‒/‒	+/+/+
B_bakery_	Bs	‒/‒/‒	‒/‒/‒	+/+/+	‒/‒/‒	‒/‒/‒	‒/‒/‒	+/+/+	‒/‒/‒
	B0	‒/‒/‒	‒/‒/‒	+/+/+	‒/‒/‒	‒/‒/‒	‒/‒/‒	+/+/+	‒/‒/‒
	B1	‒/‒/‒	‒/‒/‒	+/+/+	‒/‒/‒	‒/‒/‒	‒/‒/‒	+/+/+	‒/‒/‒
	B2	‒/‒/+	‒/‒/+	+/+/+	‒/‒/‒	‒/‒/‒	‒/‒/‒	+/+/+	‒/‒/‒
	B3	‒/‒/‒	‒/‒/+	+/+/+	‒/‒/‒	‒/‒/‒	‒/‒/‒	+/+/+	‒/‒/‒
	B4	‒/‒/‒	‒/‒/‒	+/+/+	‒/‒/‒	‒/‒/‒	‒/‒/‒	+/+/+	‒/‒/‒
C_bakery_	C0	‒/‒/‒	‒/‒/‒	‒/‒/‒	+/‒/‒	‒/‒/‒	‒/‒/‒	‒/‒/‒	‒/‒/‒
	C1	‒/‒/‒	‒/‒/‒	‒/‒/‒	+/‒/‒	‒/‒/‒	+/‒/‒	‒/‒/‒	‒/‒/‒
	C2	‒/‒/‒	‒/‒/‒	‒/‒/‒	‒/‒/‒	‒/‒/‒	‒/‒/‒	‒/‒/‒	‒/‒/‒
	C3	‒/‒/‒	‒/‒/‒	‒/‒/‒	‒/‒/‒	‒/‒/‒	‒/‒/‒	‒/‒/‒	‒/‒/‒
D_bakery_	D1	‒/‒/‒	‒/‒/‒	‒/‒/‒	‒/‒/‒	‒/‒/‒	‒/‒/‒	‒/‒/‒	‒/+/‒
	D2	‒/‒/‒	‒/‒/‒	‒/‒/‒	‒/‒/‒	‒/‒/‒	‒/‒/‒	‒/‒/‒	‒/+/+
	D3	‒/‒/‒	‒/‒/‒	‒/‒/‒	‒/‒/‒	‒/‒/‒	‒/‒/‒	‒/‒/‒	‒/+/+
	D4	‒/‒/‒	‒/‒/‒	‒/‒/‒	‒/‒/‒	‒/‒/‒	‒/‒/‒	‒/‒/‒	+/+/+
	D5	N/‒/‒	N/‒/‒	N/‒/‒	N/‒/‒	N/‒/‒	N/‒/‒	N/‒/‒	N/+/+

*Lactobacillus panis*	*Lactobacillus plantarum*	*Lactobacillus pontis*	*Lactobacillus rhamnosus*	*Lactobacillus sanfranciscensis*	*Lactobacillus secaliphilus*	*Lactobacillus vaginalis*	*Lactobacillus zymae*	*Leuconostoc lactis*	*Pantoea* sp.	*Pediococcus acidilactici*
+/+/+	‒/‒/‒	+/+/+	‒/‒/‒	‒/‒/‒	‒/‒/‒	‒/‒/‒	‒/‒/‒	‒/‒/‒	‒/‒/‒	‒/‒/‒
‒/+/+	‒/‒/‒	‒/+/+	‒/‒/‒	‒/‒/‒	‒/‒/‒	‒/‒/‒	‒/‒/‒	‒/‒/‒	‒/‒/‒	‒/‒/‒
+/+/+	‒/‒/‒	‒/+/+	‒/‒/‒	‒/‒/‒	‒/‒/‒	‒/‒/‒	‒/‒/‒	‒/‒/‒	‒/‒/‒	‒/‒/‒
+/+/+	‒/‒/‒	+/+/+	‒/‒/‒	‒/‒/‒	‒/‒/‒	+/‒/+	‒/‒/‒	‒/‒/‒	‒/‒/‒	‒/‒/‒
+/+/+	‒/‒/‒	+/+/+	‒/‒/‒	‒/‒/‒	‒/‒/‒	‒/‒/+	‒/‒/‒	‒/‒/‒	‒/‒/‒	‒/‒/‒
‒/‒/‒	‒/‒/‒	‒/‒/‒	‒/‒/‒	‒/‒/‒	‒/‒/+	‒/‒/‒	‒/‒/‒	‒/‒/‒	‒/‒/‒	‒/‒/‒
+/‒/‒	‒/‒/‒	‒/‒/+	‒/‒/‒	‒/‒/‒	‒/‒/+	‒/‒/‒	‒/‒/‒	‒/‒/‒	‒/‒/‒	‒/‒/‒
+/‒/‒	‒/‒/‒	‒/‒/‒	‒/‒/‒	‒/‒/‒	‒/‒/+	‒/‒/‒	‒/‒/‒	‒/‒/‒	‒/‒/+	‒/‒/‒
‒/‒/‒	‒/‒/‒	‒/‒/‒	‒/‒/‒	‒/‒/‒	‒/‒/+	‒/‒/‒	‒/‒/‒	‒/‒/‒	‒/‒/‒	‒/‒/‒
+/‒/‒	‒/‒/‒	‒/‒/+	‒/‒/‒	‒/‒/‒	‒/‒/+	‒/‒/‒	‒/‒/‒	‒/‒/‒	‒/‒/‒	‒/‒/‒
+/‒/‒	‒/‒/‒	‒/‒/‒	‒/‒/‒	‒/‒/‒	‒/‒/+	‒/‒/‒	‒/‒/‒	‒/‒/‒	‒/‒/‒	‒/‒/‒
‒/‒/‒	+/‒/‒	‒/+/+	+/‒/‒	‒/‒/‒	‒/‒/‒	‒/‒/‒	‒/‒/‒	‒/‒/‒	‒/‒/‒	‒/‒/‒
‒/‒/‒	‒/‒/‒	‒/+/‒	‒/‒/‒	‒/+/+	‒/‒/‒	‒/‒/‒	+/‒/+	+/‒/‒	‒/‒/‒	+/‒/‒
‒/‒/‒	‒/‒/‒	+/+/+	‒/‒/‒	‒/+/+	‒/‒/‒	‒/‒/‒	+/‒/‒	‒/‒/‒	‒/‒/‒	‒/‒/‒
‒/‒/‒	‒/‒/‒	+/+/‒	‒/‒/‒	‒/+/+	‒/‒/‒	‒/‒/‒	+/‒/+	‒/‒/‒	‒/‒/‒	‒/‒/‒
‒/‒/‒	‒/‒/‒	‒/+/+	‒/‒/‒	‒/‒/‒	‒/‒/‒	‒/‒/‒	+/+/+	‒/‒/‒	‒/‒/‒	‒/‒/‒
‒/‒/‒	‒/‒/‒	+/+/+	‒/‒/‒	‒/‒/‒	‒/‒/‒	‒/‒/‒	+/‒/‒	‒/‒/‒	‒/‒/‒	‒/‒/‒
‒/‒/‒	‒/‒/‒	+/+/+	‒/‒/‒	‒/‒/‒	‒/‒/‒	‒/‒/‒	+/‒/+	‒/‒/‒	‒/‒/‒	‒/‒/‒
‒/‒/‒	‒/‒/‒	+/+/+	‒/‒/‒	‒/‒/‒	‒/‒/‒	‒/‒/‒	+/‒/+	‒/‒/‒	‒/‒/‒	‒/‒/‒
N/‒/‒	N/‒/‒	N/+/+	N/‒/‒	N/‒/‒	N/‒/‒	N/‒/‒	N/+/‒	N/‒/‒	N/‒/‒	N/‒/‒

**Table 4:** Number of reads, OTUs, expected OTUs at 500 reads and rate of new OTUs at 500 reads obtained from 16S rRNA pyrosequencing of rye sourdoughs from four Estonian bakeries (A_bakery_, B_bakery_, C_bakery_ and D_bakery_). Samples are coded according to the description reported in Table 1.

Sample	Raw reads	Reads	OTUs	Expected OTUs at 500 reads	Rate of new OTUs at 500 reads
A0	1929	1574	15	8.745	0.008
A1	NA*	NA	NA	NA	NA
A2	4332	4153	10	4.941	0.003
A3	2740	2335	12	6.750	0.004
A4	2320	1952	22	10.561	0.011
Bs	2417	2281	7	4.643	0.001
B0	1259	303	21	NA	NA
B1	2462	1260	12	7.414	0.007
B2	859	503	18	17.940	0.020
B3	932	688	14	11.589	0.014
B4	2179	1334	17	9.875	0.010
C0	2754	2749	7	2.389	0.003
C1	1410	263	15	NA	NA
C2	1960	1922	9	4.349	0.004
C3	3569	3437	12	4.149	0.004
D1	1415	1314	13	7.186	0.009
D2	2435	2356	7	3.661	0.002
D3	991	593	11	9.878	0.012
D4	3122	2923	10	4.197	0.002
D5	3303	2966	6	2.815	0.002

* NA–not acquired

After the first sample (Bs) was collected from B_bakery_, the sourdough was renewed using fresh sourdough sourced from another bakery (sample B0). The stability of B_bakery_ sourdough was monitored for six months after renewal. The results of culture dependent analysis revealed that both *Lactobacillus amylovorus* and *Lactobacillus frumenti* were dominant species within all sourdough samples collected from B_bakery_ (Table 3 and Fig 1). The ratio between this species varied during propagation. The same fingerprint type of dominant *L*. *amylovorus* was detected throughout the study (data not shown). *L*. *panis* entered the sourdough cycle with sample B0 and remained constant within the sourdough at low counts during subsequent propagation. DGGE analysis confirmed the dominance of *L*. *amylovorus* and *L*. *frumenti* (Table 3 and S1 Fig). On the contrary, *L*. *panis* was undetectable by both DGGE and 16S rRNA pyrosequencing (Table 3 and Fig 2). Pyrosequencing analysis revealed that *L*. *frumenti* and, especially, *L*. *amylovorus*, were the dominant OTUs in the sourdough samples collected at the B_bakery_. Additional OTUs (*Lactobacillus secaliphilus* and *Lactobacillus* sp.) were found as sub-dominant in all the B_bakery_ samples. In C_bakery_, *L*. *plantarum*, *Lactobacillus rhamnosus* and *Lactobacillus casei/paracasei* were isolated from the starter sample (C0) (Fig 1). After one year of propagation (C1), *L*. *casei/paracasei* persisted and other species (*Lactobacillus zymae*, *L*. *fermentum*, *Leuconostoc lactis* and *Pediococcus acidilactici*) were detected. In the sourdough samples taken 21 and 28 months after renewal (samples C2 and C3), all the LAB species previously detected, except for *L*. *zymae*, seemed to be replaced by *L*. *pontis*. In contrast, DGGE analysis revealed *L*. *pontis* as the only species in sample C0 and *L*. *pontis* and *L*. *sanfranciscensis* in all the remaining sourdough samples (Table 3 and S1 Fig). Overall, pyrosequencing analysis of C_bakery_ samples was in agreement with DGGE, excluding the lack of *L*. *pontis* in C1 and C3 samples and the presence, at high relative abundance, of *L*. *zymae* in C1 (Table 3 and Fig 2). Given the discrepancy between culture dependent and independent analyses regarding the presence of *L*. *sanfranciscensis* in the samples collected at the C_bakery_, the C3 sample was analyzed using two additional media, SDB and mMRS. *L*. *sanfranciscensis* could be isolated after an extended incubation time (72 h) of mMRS plates (data not shown).

The sourdough samples from D_bakery_ contained *L*. *zymae*, *L*. *pontis* and *L*. *helveticus* (Figs 1 and 2 and S1 Fig). The relative proportion of these species in a given sourdough sample depended on the ambient temperature in the bakery. In the wintertime (sample D1), growth of *L*. *zymae* was favored, while *L*. *pontis* and *L*. *helveticus* dominated in the samples (D2, D3, and D4) collected during warmer periods.

### Impact of new propagation parameters on the LAB community of D_bakery_ sourdough

**Fig 3:**
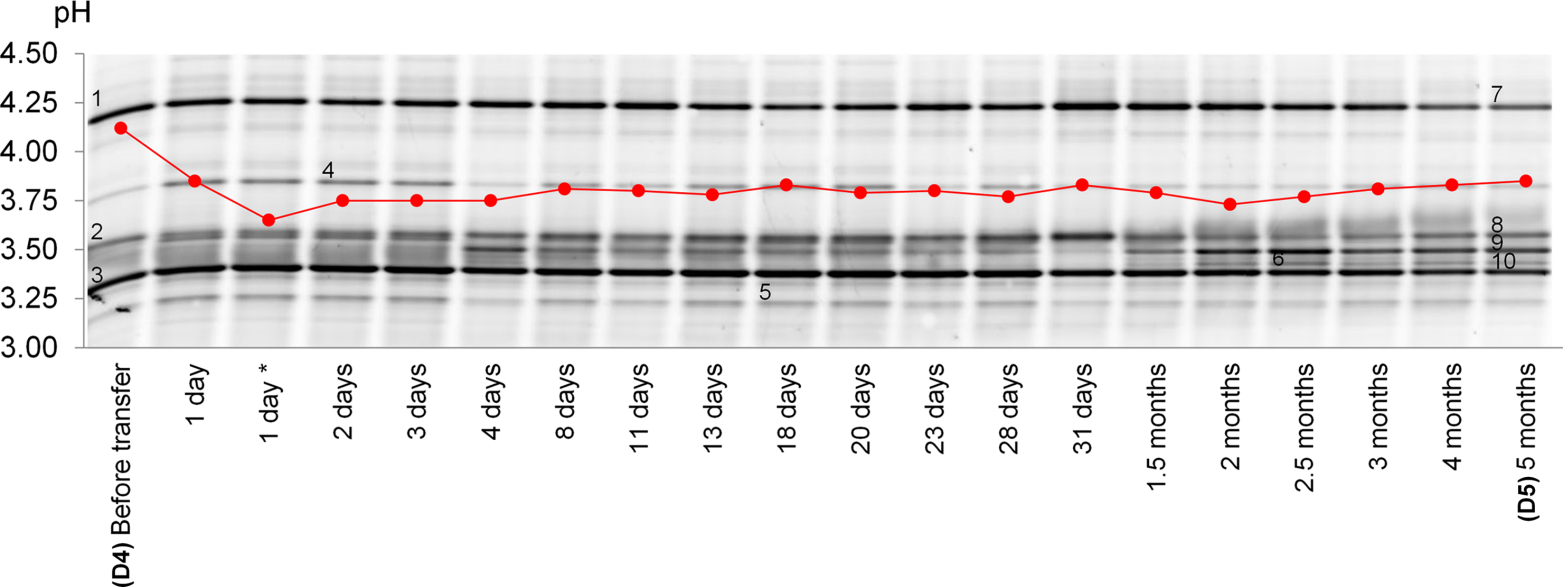
pH (red plot) and lactic acid bacterium species detected by DGGE analysis of the 16S rRNA gene amplicons in rye sourdoughs collected at the D_bakery_ before (D4) and after applying the new propagation protocol. Time after transfer is indicated below the gel (d–day; m–month). Bands: 1, 7 –*Lactobacillus helveticus*; 2, 8 –Cereal chloroplast DNA; 3, 4, 5, 10 –*Lactobacillus pontis*; 6 –*Lactobacillus* sp.; 9 –*Lactobacillus zymae*; *—sample collected from sourdough after 36 h storage at 5°C.

In order to improve the stability of D_bakery_ sourdough, a new sourdough propagation protocol was applied with a controlled fermentation temperature, prolonged fermentation time and reduced inoculum size. As ascertained through culture independent analyses, *L*. *pontis* and *L*. *helveticus* species dominated in the sourdough (sample D5) after 5 months of propagation performed under the new protocol (Figs 2 and 3). The pH of the sourdough ranged from 3.73–3.79 during the five months of observation (Fig 3).

### Analysis of yeast communities in rye sourdoughs

DGGE analysis of amplified 28S rRNA (S2 Fig) was performed to identify the yeast species in the sourdoughs collected at the four bakeries. *Kazachstania telluris* was the only yeast species harbored in the freeze-dried starter (A0), as well as in all the sourdough samples collected at the A_bakery_. No yeast species were detected in B_bakery_ sourdough. *Candida humilis* was the only yeast species detected in C_bakery_ and D_bakery_ sourdough samples, whereas no yeast species were detected in the starter (C0) used in C_bakery_ (S2 Fig).

### Beta-diversity across the sourdough samples

**Fig 4:**
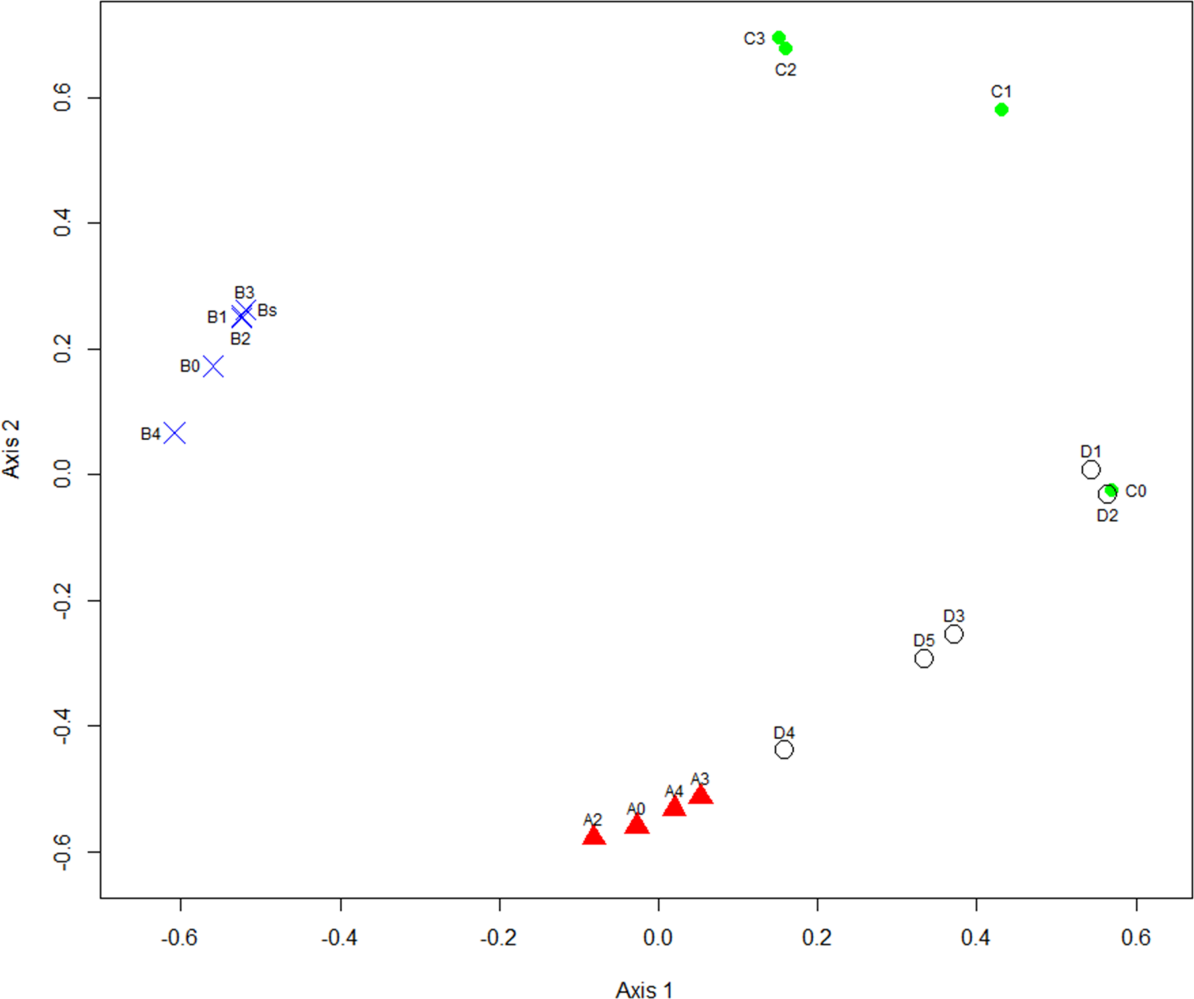
Two dimensional non-metric multidimensional scaling (NMDS) of sourdough samples from four Estonian bakeries (A_bakery_, B_bakery_, C_bakery_ and D_bakery_).

Beta-diversity analysis was performed on the pyrosequencing data to compare diversity between each sourdough sample and to determine the similarity (or difference) in species composition of the samples (Fig 4). Two-dimensional non-metric multidimensional scaling (NMDS) provided a stress value of 0.156 and an R2 value of 0.885. The stress value decreased to 0.064 and the R2 value increased to 0.977 when calculating the NMDS with three dimensions. Sourdough samples from A_bakery_ and B_bakery_ grouped in two different clusters, both characterized by closeness of the grouped samples, which illustrates the stability of both sourdough propagation processes. On the contrary, samples from both C_bakery_ and, especially, D_bakery_ grouped in looser clusters, thus indicating that the bacterial communities within these sourdoughs are less stable (Fig 4). The freeze-dried starter C0 that contained *L*. *pontis* groups together with D_bakery_ samples where *L*. *pontis* is prevalent. A_bakery_ and D_bakery_ sourdough samples can be found in the same quadrant of the NMDS plot because both contain *L*. *helveticus* and *L*. *pontis*. Samples collected at the B_bakery_ which employs a higher fermentation temperature, differ significantly from all other sourdoughs.

## Discussion

This study evaluates the stability of both the microbial communities and chemical properties of rye sourdoughs from four Estonian bakeries. The bakeries used flour from the same source, but employed different propagation parameters. Our data showed that controlling the propagation conditions stabilized the cell density and distribution of prevalent LAB species in rye sourdoughs during long term propagation. The cell density of culturable LAB fluctuated in sourdoughs fermented at ambient temperature. High LAB cell densities correlate with high titratable acidity and low pH, which are both characteristic of mature rye sourdough and prerequisite for producing rye bread with desirable sensory properties. B_bakery_ sourdough samples showed the highest acidity because of the highest fermentation temperature. In contrast, samples collected in both C_bakery_ and D_bakery_ were characterized by insufficient acidity during the winter, probably due to the combination of low ambient temperature and short fermentation cycle. Adoption of a constant fermentation temperature (30°C) by D_bakery_ resulted in the stabilization of acid production and LAB cell density, even in this small scale bakery.

Higher stability of LAB communities was found in sourdough fermented at controlled conditions, compared to those fermented at ambient temperature that seemed strongly affected by the season of collection. Generally, the number of cycles of propagation of type II sourdoughs is lower than type I sourdough due to instability of the microbial community. The starter bacteria are often outcompeted by microorganisms contaminating flour and bakery environment. However, the sourdoughs collected in both large-scale bakeries (A_bakery_ and B_bakery_) considered in this study showed better stability. This is probably due to the starter preparation chosen, which contained LAB communities that had been previously adapted to the propagation parameters employed in these bakeries. *L*. *helveticus* alone or *L*. *amylovorus* together with *L*. *frumenti* were the dominant LAB species in A_bakery_ and B_bakery_, respectively. *L*. *amylovorus* and *L*. *frumenti* were previously identified as prevalent in other industrial rye sourdoughs propagated at elevated fermentation temperatures [4–5], similar to that (42–44°C) applied in B_bakery_, and are characterized by strong thermo- and acid-tolerance [27]. The persistence of *L*. *amylovorus* in sourdoughs may be also attributed to its high amylolytic activity and ability to produce bacteriocine amylovorin, a common feature for representatives of this species [28–29]. The same fingerprint type of dominant *L*. *amylovorus* was detected throughout the study, including the fresh sourdough (B0) from another bakery belonging to the same corporation. Adaptation to the sourdough environment could be the reason for such remarkable stability.

Although *L*. *helveticus* is not a common dominant species in sourdoughs [4, 13, 16] we found it as dominant bacterial species also in the sourdough of the small scale D_bakery_. However, in contrast with A_bakery_, the sourdough fermentation at ambient temperature in D_bakery_ prevented the stable prevalence of thermophilic *L*. *helveticus*. Indeed, depending on the season, *L*. *pontis* and *L*. *zymae* prevailed over *L*. *helveticus*. *L*. *zymae*, a species capable of growing at lower temperatures, has previously been found in both Greek and Belgian wheat sourdoughs, which indicates that it is widely spread [30–31]. New propagation parameters (higher fermentation temperature, decreased inoculum size, prolonged fermentation time, and use of 4°C refrigeration during breaks in production) adopted in D_bakery_ stabilized the LAB community and triggered an increase in the proportion of *L*. *helveticus* and *L*. *pontis*. No new species originating from raw materials or bakery environment were detected in the sourdough community of D_bakery_ even after five months of using the new protocol. This suggests high competitiveness and robustness of the dominant LAB that had adapted to different temperatures and initial sourdough acidity, although the house microbiota of the bakery may have also been the source of these LAB. The importance of house microbiota in the stability of sourdough microbial communities has been shown [12, 32].

Representatives of *L*. *zymae* and *L*. *pontis* species were also detected among the dominant population of LAB in C_bakery_, which utilized sourdough propagation parameters that are very similar to those originally applied in D_bakery_. Unfortunately, comparing representatives of *L*. *pontis* with those contained in the commercial starter used in this bakery was not possible since no *L*. *pontis* was isolated. In contrast with the D_bakery_ sourdough, *L*. *sanfranciscensis* was also identified among prevailing bacteria in sourdough samples from C_bakery_. *L*. *sanfranciscensis* is frequently found in type I sourdoughs due to its adaptation to sourdough conditions, its small genome, and metabolism [4]. Stable non-competitive association of this maltose-positive LAB species with maltose-negative yeast *C*. *humilis* exists in traditional sourdoughs [33]. *L*. *sanfranciscensis* species is capable of hydrolyzing maltose by intracellular maltose phosphorylase activity and thereby accumulate glucose in the environment for *C*. *humilis* to utilize [34]. *C*. *humilis* was the only yeast species identified in the sourdoughs of both small-scale productions C_bakery_ and D_bakery_.

Co-existence of *L*. *helveticus* with the yeast species *K*. *telluris* was found in the sourdough samples collected at the A_bakery_. *K*. *telluris* (formerly *Saccharomyces telluris*, *Arxiozyma telluris*) is mainly known to cause infections in rodents and it may be isolated from soil [35]. Occurrence of this species in sourdough has not been previously reported. However, this thermophilic yeast is able to ferment glucose and grow on glucose, ethanol, and lactic acid [35]. As our identification is based only on culture-independent method (sequencing of 28S rRNA), further research should be carried out to assess the role of this yeast species in the sourdough community. The high fermentation temperature in B_bakery_ prevented the development of yeasts in the sourdough.

The culture independent methods applied in this study enabled us to identify LAB species (e.g. *L*. *secaliphilus* and *L*. *sanfranciscensis*) from sourdough and starter samples that were difficult to be cultivated. It has been previously shown that many sourdough LAB are sensitive to oxygen and/or have complex nutrient requirements [27, 36–37]. A wide variety of media should therefore be used to isolate sourdough LAB, since there is no universal medium that is suitable for all LAB. Culture independent methods such as DGGE and pyrosequencing enable one to detect LAB that are difficult to culture on common laboratory media. High throughput sequencing also allows for species identification at the sub-population level and provides quantitative information regarding the relative abundance of species within sourdough [16, 38].

Our data showed that sourdough bacterial communities within large-scale production facilities can be stable for many months using controlled propagation conditions, whereas, fermentation at room temperature leads to seasonal fluctuations in the species composition.

## Supporting Information

S1 Fig Lactic acid bacterium species detected by DGGE analysis of the 16S rRNA gene amplicons in rye sourdoughs from four Estonian bakeries (A_bakery_, B_bakery_, C_bakery_ and D_bakery_). **Samples are coded according to the description reported in Table 1.** Bands: 1 –*Lactobacillus helveticus*; 2 –*Lactobacillus panis*; 3 –Cereal chloroplast DNA; 4 –*Lactobacillus pontis*; 5 –*Lactobacillus amylovorus*; 6 –Cereal chloroplast DNA; 7 –*Lactobacillus frumenti*; 8–10 –*Lactobacillus pontis*; 11 –Cereal chloroplast DNA; 12 –*Lactobacillus pontis*; 13 –*Lactobacillus sanfranciscensis*; 14 –*Lactobacillus pontis*; 15 –*Lactobacillus helveticus*; 16 –*Lactobacillus pontis*; 17 –Cereal chloroplast DNA; 18 –*Lactobacillus zymae*; 19 –*Lactobacillus pontis*. Samples are coded according to the description reported in Table 1.
(TIF)

S2 Fig Yeast species detected by DGGE analysis of the 28S rRNA gene amplicons in rye sourdoughs from three Estonian bakeries (A_bakery_, C_bakery_ and D_bakery_). Bands: 1, 2 –*Kazachstania telluris*; 3 –Cereal DNA; 4, 5, 6, 7 –*Candida humilis*. Samples are coded according to the description reported in Table 1.
(TIF)
