# Supplementary figures and images for: Diversity and Stability of Lactic Acid Bacteria in Rye Sourdoughs of Four Bakeries with Different Propagation Parameters
Source: PLoS One. 2016 Feb 5;11(2):e0148325. doi: 10.1371/journal.pone.0148325 (PMC4743960; doi:10.1371/journal.pone.0148325)

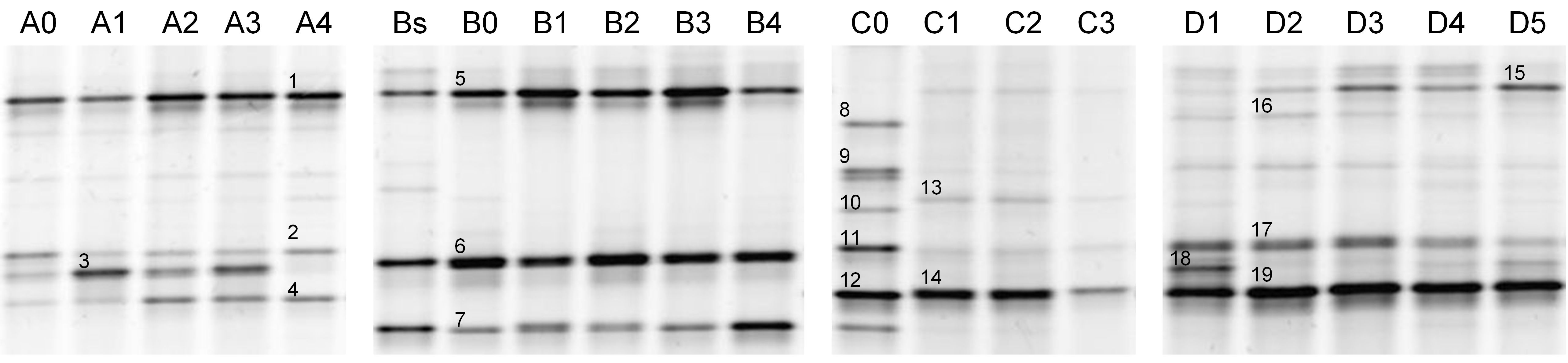

Supplement: S1 Fig — Samples are coded according to the description reported in Table 1. Bands: 1 –Lactobacillus helveticus; 2 –Lactobacillus panis; 3 –Cereal chloroplast DNA; 4 –Lactobacillus pontis; 5 –Lactobacillus amylovorus; 6 –Cereal chloroplast DNA; 7 –Lactobacillus frumenti; 8–10 –Lactobacillus pontis; 11 –Cereal chloroplast DNA; 12 –Lactobacillus pontis; 13 –Lactobacillus sanfranciscensis; 14 –Lactobacillus pontis; 15 –Lactobacillus helveticus; 16 –Lactobacillus pontis; 17 –Cereal chloroplast DNA; 18 –Lactobacillus zymae; 19 –Lactobacillus pontis. Samples are coded according to the description reported in Table 1. (TIF) [file pone.0148325.s001.tif]

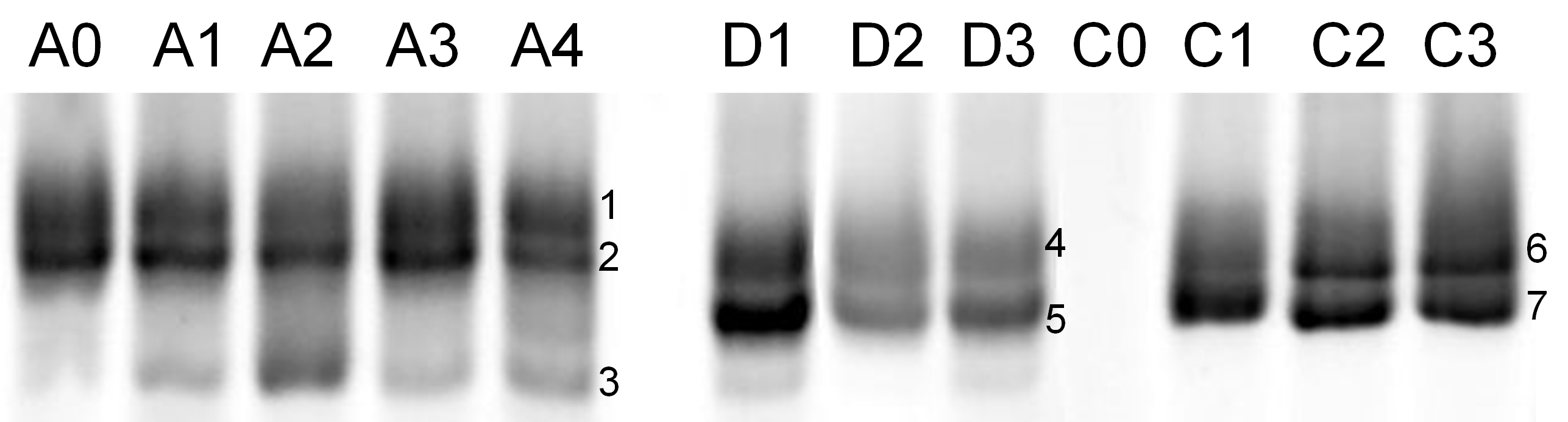

Supplement: S2 Fig — Bands: 1, 2 –Kazachstania telluris; 3 –Cereal DNA; 4, 5, 6, 7 –Candida humilis. Samples are coded according to the description reported in Table 1. (TIF) [file pone.0148325.s002.tif]
